# Spatiotemporal heterogeneity of gestational syphilis in the 1st Health Region of Pará in the Brazilian Amazon

**DOI:** 10.1590/1414-431X2026e15574

**Published:** 2026-07-03

**Authors:** K.R. Silva, R.G. Silva, R.J.P.S. Guimarães, C.R. Mesquita, L.M.V. Nogueira, M.E.C. Rassy

**Affiliations:** 1 Programa de Pós-Graduação em Enfermagem, Universidade do Estado do Pará, Belém, PA, Brasil; 2 Instituto Evandro Chagas, Secretaria de Vigilância em Saúde e Ambiente, Ministério da Saúde, Ananindeua, PA, Brasil; 3 Programa de Pós-Graduação em Enfermagem, Universidade Federal do Pará, Belém, PA, Brasil

**Keywords:** Gestational syphilis, Spatiotemporal analysis, Amazon

## Abstract

Syphilis in pregnant women remains an important public health problem in Brazil, especially in urban contexts marked by social inequalities and weaknesses in the organization of health services. In the Amazon region, territorial, socioeconomic, and healthcare-related characteristics influence access to prenatal care, timely diagnosis, and the control of vertical transmission. Thus, this study aimed to analyze the spatiotemporal patterns of syphilis among pregnant women in a health region of the Brazilian Amazon. This is an ecological, descriptive, and quantitative study that evaluated 5,607 positive cases of syphilis in pregnant women residing in the 1st Health Region (Metropolitan I) of Pará, composed of Belém, Ananindeua, Marituba, Benevides, and Santa Bárbara, reported between 2019 and 2024. Data were obtained from the Pará State Health Department, and the “geocodebr” package from the National Institute for Space Research and the R software were used for georeferencing the geographic coordinates of each case. Kernel Density Estimation analysis identified the presence of high-density clusters in the 2019-2020 and 2023-2024 biennia. Spatial scan analysis identified Marituba as a significant cluster, indicating higher risk and a constant concentration of cases throughout the analyzed period. Syphilis in pregnant women presents a heterogeneous and persistent spatiotemporal pattern in the region, requiring integrated, territorialized, and intersectoral strategies.

## Introduction

Syphilis is caused by *Treponema pallidum*, a systemic infection that can lead to cutaneous, bone, cardiovascular, and neurological lesions, and even death. The manifestations of infection vary with progression and stage and may be classified as primary, secondary, latent, or tertiary (1).

Sexual contact with a symptomatic or asymptomatic individual without the use of female or male condoms may result in transmission and is considered the main route (1-3). Transmission may also occur vertically during pregnancy or childbirth. In such cases, early identification and treatment of the infection are essential to prevent harm to the child. In this context, gestational syphilis (GS) becomes particularly relevant in the epidemiological scenario of the disease (4).

In 2024, the detection rate of GS in Brazil was 35.4 per thousand live births; in the Northern region, 31.7 per thousand live births; in the state of Pará, 30.2 per thousand live births; and in the capital Belém, 56.3 per thousand live births (1). Among the 89,724 cases recorded in Brazil, 49.3% of pregnant women were diagnosed in the first trimester of pregnancy and 19.9% in the second trimester, which represents a favorable period to strategically initiate therapeutic planning to prevent vertical transmission (1). Therefore, prenatal care follow-up is a priority for identifying GS cases; however, although 98% of Brazilian pregnant women receive prenatal care, at least 10% do not undergo even one serological test for syphilis, which contributes to late diagnosis and directly impacts the timely implementation of treatment (5).

Pregnant women diagnosed with syphilis are often inserted into contexts of health vulnerability, in which insufficient access to prevention resources, timely diagnosis, and adequate treatment constitutes a violation of second-generation fundamental rights, especially regarding the right to health. Regional inequalities in Brazil are known to influence access to health services and may compromise prenatal care in certain locations, resulting in unsatisfactory neonatal outcomes (4,6,7).

The Brazilian Amazon region presents particular environmental, social, economic, and geographic characteristics that shape access to health services and the implementation of public policies. In this context, comprehensive knowledge of territories and their specificities becomes essential. Understanding the epidemiological dynamics involving GS is therefore fundamental for developing effective public policies, especially in regions with social, economic, and territorial particularities such as the Amazon (8,9). Consequently, regionalization emerges as a strategy that uses spatial divisions to plan, organize, and manage health action and service networks, enabling users from different regions to access care within a satisfactory timeframe (10).

Thus, analyzing the spatial distribution of GS cases allows the identification of case concentration, spatial patterns, and areas of greater vulnerability that require increased attention for the development of equitable health actions. Moreover, time-series analysis of cases enables the identification of trends and seasonal variations that may influence disease incidence (6,11,12).

In this sense, to better understand the context surrounding GS, it is necessary to examine how geographic space influences GS cases and their repercussions. Therefore, the analysis of the spatial distribution of GS cases may support targeted actions that contribute to more favorable outcomes.

Thus, this study aims to analyze the spatiotemporal patterns of GS in territorial health units within the Brazilian Amazon context.

## Material and Methods

This is an ecological, descriptive study with a quantitative approach conducted in the state of Pará, aimed at supporting the planning of health actions, decentralization of services, and reduction of geographic barriers to improve users' access to health care.

### Study area

Pará is divided into 13 Health Regions, and the study was conducted in the 1st Health Region (Metropolitan I), composed of the municipalities of Belém, Ananindeua, Marituba, Benevides, and Santa Bárbara. The estimated population of this region in 2025 was 2,117,471 inhabitants, with a Human Development Index (HDI) ranging from 0.746 to 0.627 (13,14).

### Data collection

The study population consisted of all positive cases of GS from 15 years of age onwards, recorded between 2019 and 2024, reported in the Notifiable Diseases Information System (*Sistema de Informação de Agravos de Notificação* - Sinan), and made available by the Pará State Health Department (SESPA). A total of 5,608 SG cases were identified, and one case was excluded because the pregnant woman was not a resident of the 1st Health Region. Thus, 5,607 SG cases were eligible for the study.

### Data analysis

The database obtained was organized into spreadsheets using Microsoft Office Excel^®^ 2024, and data cleaning procedures were performed to ensure greater consistency and completeness and to reduce redundancy. Subsequently, georeferencing/geocoding of the database was performed using the “geocodebr” package from the National Institute for Space Research (INPE) with the support of the R software, generating geographic coordinates for each case. The result of the georeferencing process was the Geographic Database (GDB).

Using the GDB, the socioepidemiological profile was obtained based on variables from the Gestational Syphilis notification form (https://portalsinan.saude.gov.br/sifilis-em-gestante), such as: Notification Date, Municipality of Notification, Health Unit, Date of Diagnosis, Date of Birth, Age, Sex, Race/Color, Municipality of Residence, Area, Occupation, and Education. The following epidemiological variables were also selected: Prenatal Care during this pregnancy, Municipality where prenatal care is performed, Maternal syphilis diagnosis, Clinical Diagnosis, Presence of signs and symptoms, Treatment regimen, and Case outcome.

Based on the information obtained, descriptive statistical analysis was performed using absolute and relative frequency tables. To reduce annual rate fluctuations, stratification by biennia was carried out, allowing greater stability of measures and better interpretation of the temporal dynamics of the condition in the region, using the Statistical Package for the Social Sciences (SPSS) version 25.

The chi-squared test was applied to qualitative variables, and Pearson's correlation test was applied to quantitative variables, considering a P-value ≤0.05 for all statistical tests. In addition, the detection rate of GS per 1,000 live births was calculated, using the number of confirmed cases as the numerator and live births from the same period and municipality as the denominator. The incidence rate was calculated using the number of GS cases per year divided by the estimated population of the same year multiplied by 100,000. Live birth data were obtained from the Live Births Panel [http://plataforma.saude.gov.br/natalidade/nascidos-vivos/]. Finally, an interrupted time series analysis was employed to assess trends in detection coefficients over the years, observing possible structural changes in the temporal trajectory of the condition.

Spatial analyses were performed, including spatial distribution of GS cases, choropleth maps of GS incidence rates by year, Global Moran's Index (GMI) to identify the presence of spatial autocorrelation (spatial pattern), Kernel Density Estimation (KDE) to verify the presence of clusters, and Spatial scan statistics (Scan) to detect significant spatiotemporal clusters of GS cases.

The classes of the choropleth maps were obtained by quartiles and divided into five categories: no cases (white), low (green), medium (yellow), high (orange), and very high (red). KDE used an adaptive radius and was applied to GS detection rate data. The GMI was applied with 999 permutations. The Scan analysis was performed using SaTScan 10.3.2 and applied the discrete Poisson probabilistic model. Spatial distribution, choropleth maps, GMI, and KDE were generated using ArcGIS 10.4 software.

### Ethics committee

This study followed the principles of Resolution 466/12 of the National Health Council (CNS) of the Brazilian Ministry of Health regarding research involving human beings, as well as Resolution 510/16, which establishes standards for research in human and social sciences whose methodological approaches involve the use of restricted-access information and records. The project was approved by the Research Ethics Committee (REC) involving Human Beings of the Universidade do Estado Pará (UEPA) under protocol number 7,860,631.

## Results

A total of 5,607 GS cases were analyzed between 2019 and 2024. A progressive increase in positive cases was observed over the years, with a higher concentration in the 2023-2024 biennium, especially in 2024, which accounted for 24.7% of positive cases, indicating a recent intensification of the condition in the region (Supplementary Table S1).

In Supplementary Table S1, the annual distribution showed heterogeneity among municipalities (P<0.001), with Belém and Marituba concentrating the highest absolute number of cases. Additionally, 57.8% were diagnosed in the third trimester of pregnancy, indicating late detection. The distribution showed statistically significant differences among municipalities (P<0.001), with higher proportions of early diagnosis in Benevides and Santa Bárbara do Pará.

Moreover, the mean age of pregnant women was 23.8 years (SD=5.7), with significant variation among municipalities (P=0.001), with Santa Bárbara do Pará presenting the lowest mean age, 21.2 years (SD=4.8). Furthermore, the study population was predominantly mixed-race (83.6%), with statistically significant differences among municipalities (P<0.001), and resided in urban areas (98%). Regarding municipality of residence, Belém accounted for 63.5% of cases, followed by Ananindeua (21.2%). In terms of education, women with a high school diploma predominated (30.9%), also with significant differences among municipalities (P<0.001).

Regarding prenatal care, 48% of pregnant women were followed up in Belém, although 22.6% had no record of the municipality where prenatal care was performed. This distribution varied significantly among municipalities of residence (P<0.001), indicating care flows concentrated in larger urban centers.

In Supplementary Table S2, concerning clinical aspects, primary syphilis was the most frequent (39.4%), followed by tertiary (30.5%) and latent (23.5%), indicating a high proportion of advanced clinical presentations with statistically significant differences among municipalities (P<0.001) (Supplementary Table S2). Among the tests performed, 87.3% of pregnant women had reactive non-treponemal tests and 71.4% had reactive treponemal tests, although there was a considerable proportion of tests not performed, especially the treponemal test (23.5%). It is noteworthy that the most frequently used treatment was benzathine penicillin G 7.2 million IU (67.4%). A significant difference was observed in the distribution of treatment regimens among municipalities (P<0.001) (Supplementary Table S3).

Regarding partner management, low adherence to treatment was observed: 58.3% were not treated, with statistically significant variation among municipalities (P=0.021). Among those who received intervention, the 7.2 million IU regimen predominated (38.4%). Among the reasons for absence of treatment, “other reason” (45.6%), lack of contact with the pregnant woman (26.5%), and failures in communication or service outreach (12.8%) stood out, with significant differences among municipalities (P<0.001).

**Figure 1:**
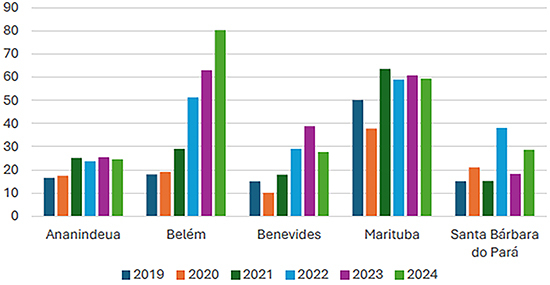
Interrupted time series of gestational syphilis cases per 1,000 live births, 2019-2024.

The time series (Figure 1) showed an increasing trend in the GS rate throughout the study period in the municipality of Belém, suggesting a continuous rise in occurrence and possible impacts of care discontinuities in 2020. However, Marituba presented high rates throughout the series, while Ananindeua maintained rates below 30 cases per thousand live births throughout the period.

**Figure 2:**
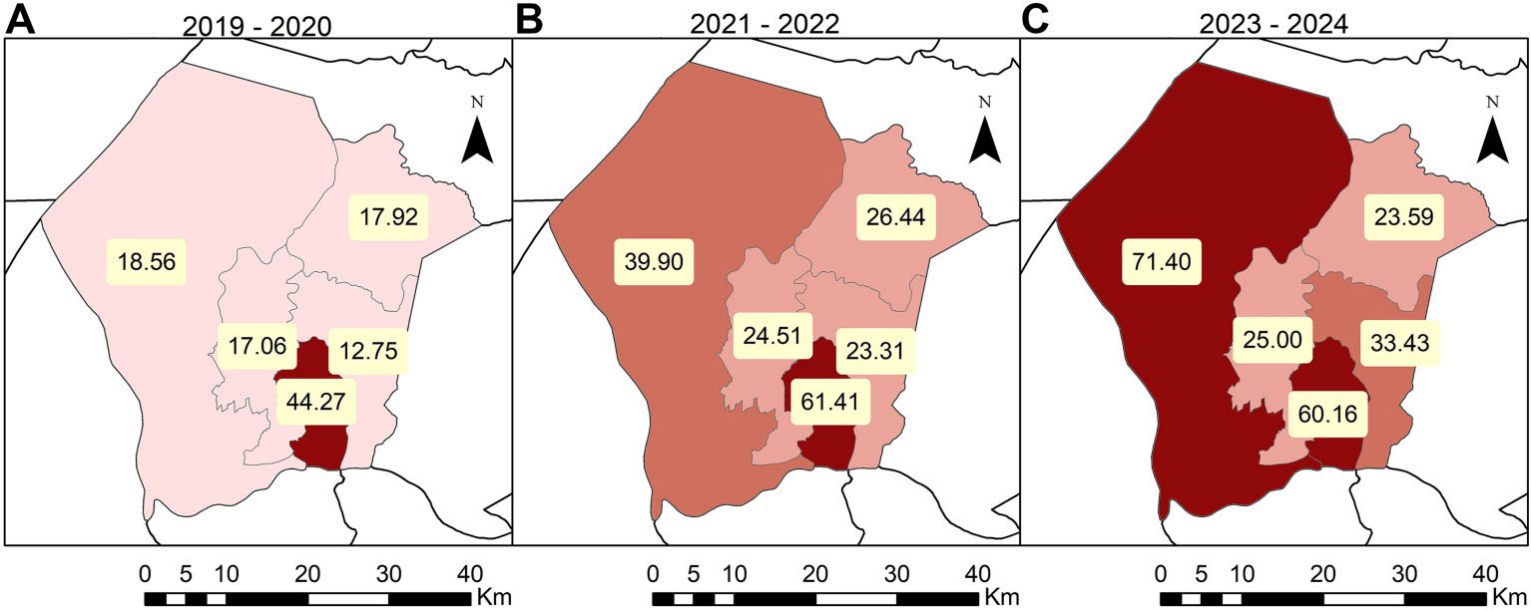
Spatiotemporal distribution of gestational syphilis rates (per 1,000 live births) by biennium, **A**, 2019-2020; **B**, 2021-2022; **C**, 2023-2024. Belém, PA, Brazil, 2019-2024 (n=5,607).

The spatiotemporal analysis (Figure 2) showed concentration of cases in the municipality of Marituba, with intensification across biennia (44.27; 61.41; and 60.16, respectively in the 2019-2020, 2021-2022, and 2023-2024 biennia).

Figure 2B shows an overall increase in case concentration in the study area and Figure 2C presents the highest density identified in the municipality of Belém (71.4).

**Figure 3:**
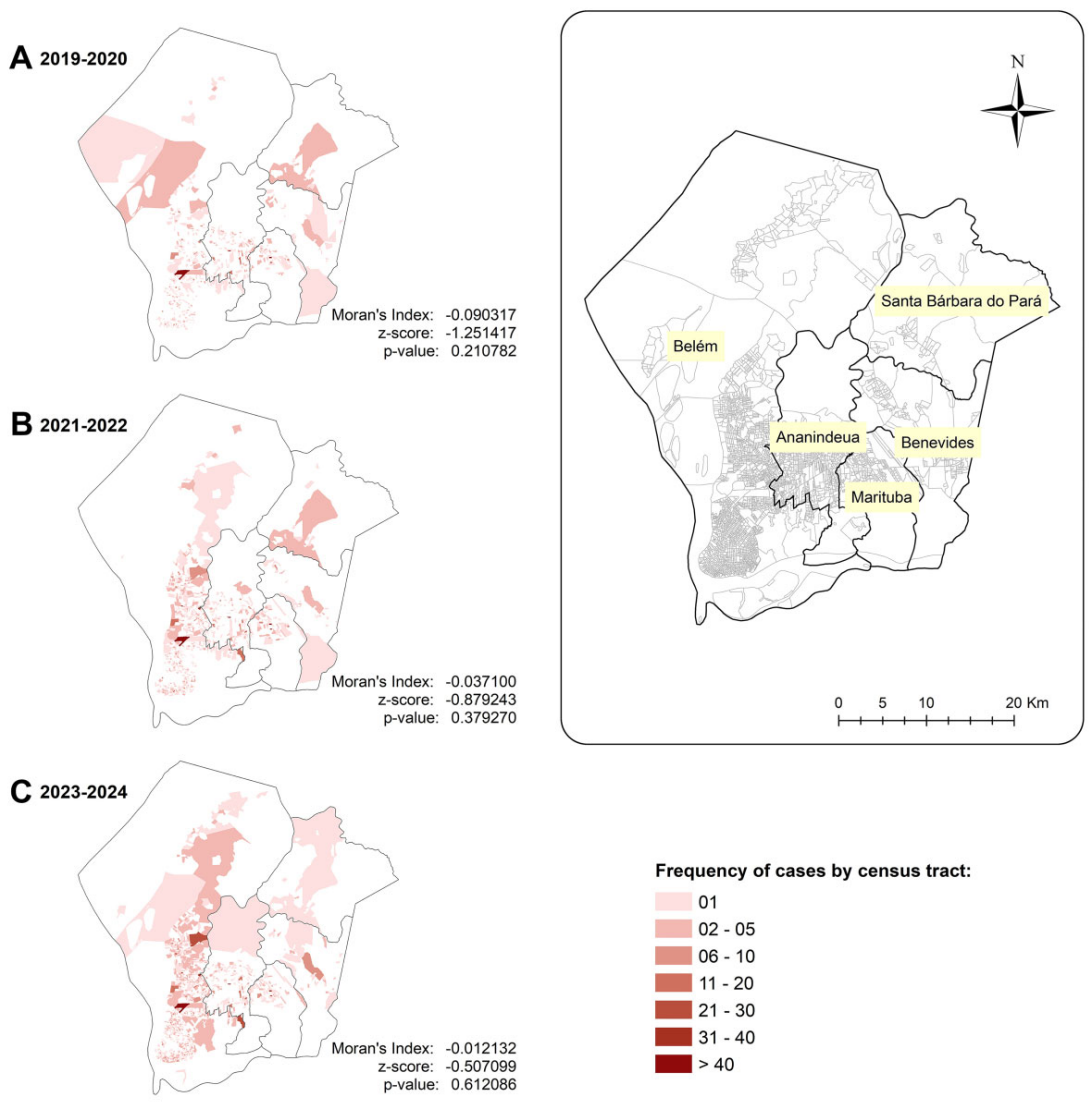
Spatial distribution of gestational syphilis cases by census tracts and biennia. 1st Health Region (Metropolitan I), Pará, Brazil, 2025. Moran’s Global Index was used for statistical analysis.

Figure 3 presents the choropleth map of GS cases across municipalities and the GMI, stratified by biennia. The panels show a gradual increase in the number of sectors with darker coloration over time, indicating expansion of notifications in the territory. The legend indicates that most sectors concentrate between 1 and 10 cases, while few present values above 40 records. The panel on the right illustrates the detailed urban grid of the municipalities in the region, showing that most cases are located in densely populated urban areas, especially in Belém and neighboring municipalities. These findings demonstrate heterogeneous and dispersed spatiotemporal growth of GS in the metropolitan region of Pará.

**Figure 4:**
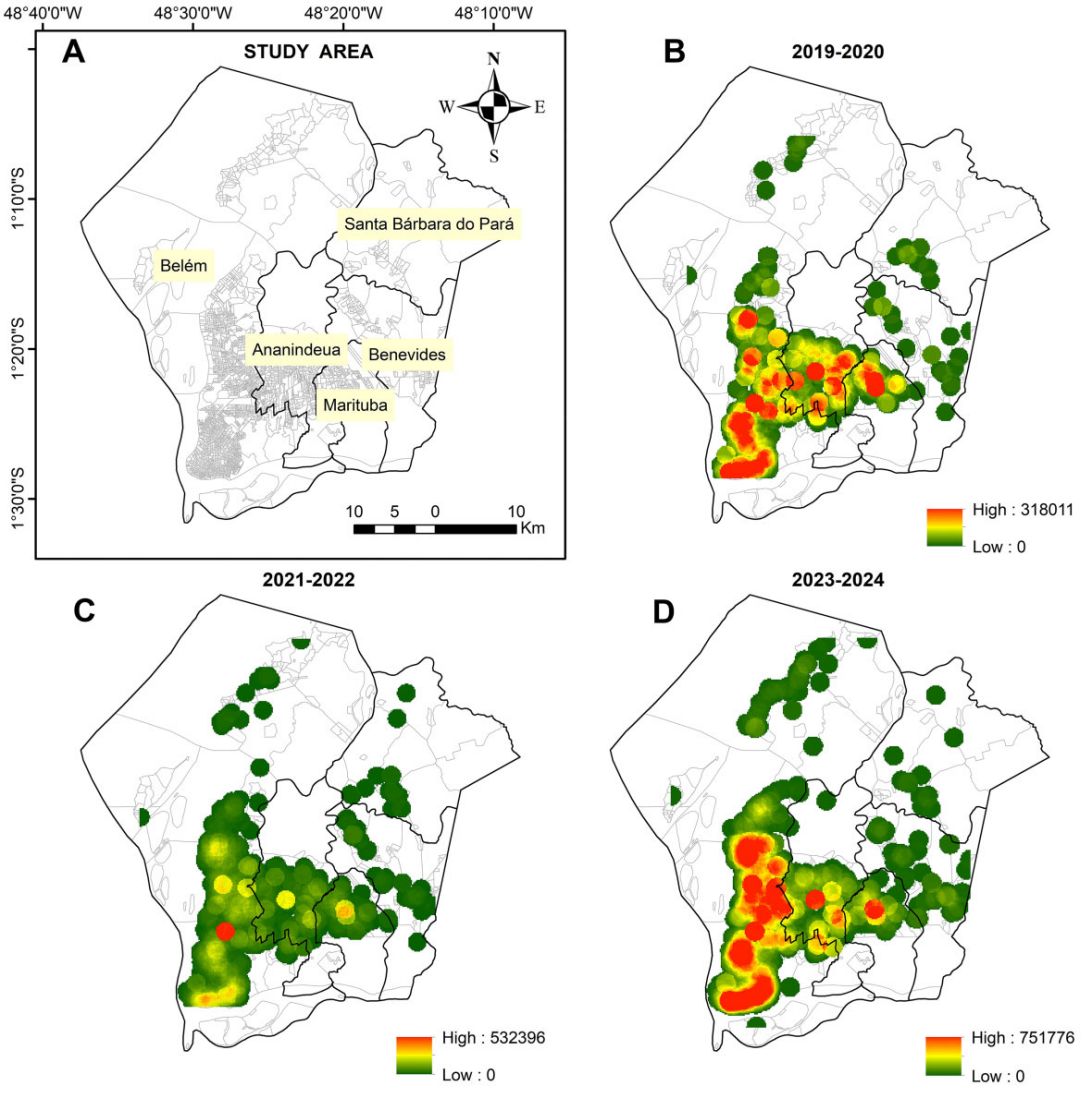
Kernel Density Estimation (KDE) map. **A**, Study area; **B**, KDE applied to the 2019-2020 biennium; **C**, KDE applied to the 2021-2022 biennium; **D**, KDE applied to the 2023-2024 biennium.

The application of KDE (Figure 4) identified high-density clusters in the 2019-2020 (Figure 4B) and 2023-2024 (Figure 4D) biennia in the municipalities of Belém, Ananindeua, and Marituba. A high-density cluster was also observed in Belém during the 2021-2022 biennium (Figure 4C).

The retrospective spatial scan analysis of syphilis cases using the discrete Poisson probabilistic model identified the municipality of Marituba with an estimated relative risk of 1.73 and a P-value lower than 0.01, indicating a significant concentration of cases during the analyzed period.

## Discussion

The clustering of cases identified in urban centers is strongly associated with the simultaneous presence of factors such as urban poverty, intense population mobility, and increased susceptibility to sexually transmitted infections (STIs). This scenario particularly affects younger pregnant women, who generally have a greater propensity for risk behaviors, including late initiation of prenatal care and barriers to adhering to preventive guidance, which contributes to the continuity of syphilis transmission in these areas (15). In the Amazonian context, these vulnerabilities are intensified by barriers to accessing health services and marked disparities among cities (16).

Furthermore, the significant increase in rates in the municipality of Belém, together with the high coefficients observed in Marituba, suggests that even with more available services and expanded testing, these actions are still insufficient to interrupt transmission. This situation reflects challenges in health surveillance, since increased notifications may indicate both greater detection and limitations in the effectiveness of prevention and care actions (15,17). In this context, studies conducted in Brazil and other Latin American countries show that expanding testing alone, without rapid diagnosis, adequate treatment, and partner involvement, tends to have a limited impact on reducing GS cases (18-21).

Moreover, the late diagnosis of syphilis, predominantly identified in the third trimester of pregnancy, represents one of the most significant findings of this research. This pattern highlights limitations in early screening during prenatal care, even among pregnant women with intermediate educational levels, such as those who completed high school education. This suggests that schooling does not necessarily translate into adequate health literacy or improved ability to navigate health services. Evidence in the literature indicates that identifying syphilis after the second trimester considerably reduces the effectiveness of interventions to prevent vertical transmission, even when treatment is initiated before delivery (22-25). Thus, late diagnosis reflects weaknesses in early prenatal screening, including delayed initiation of follow-up, insufficient number of consultations, and errors in repeating serological tests according to current recommendations (26,27).

Additionally, the high proportion of advanced clinical forms, such as tertiary syphilis, reinforces the hypothesis that many STIs remain undiagnosed or untreated at the appropriate time, while also suggesting possible failures in the interpretation of serological tests and in adequate clinical management of cases (28,29). Although the predominance of primary syphilis may indicate greater sensitivity of surveillance systems, the presence of more advanced stages reveals considerable gaps in continuity of care and integration between epidemiological surveillance and prenatal assistance (30).

Regarding care-related aspects, it was observed that despite the high performance of non-treponemal tests, a relevant proportion of pregnant women lacked confirmation by treponemal testing. This finding is consistent with national studies pointing to operational barriers in the availability of confirmatory tests, particularly in Primary Health Care settings located in peripheral areas (1,31). The absence of treponemal testing compromises diagnostic accuracy, favors inappropriate treatments, and hinders monitoring of therapeutic response, thereby negatively impacting GS control (1,31).

Another relevant aspect highlighted by this study is the low adherence to treatment among sexual partners, observed in more than half of the cases. The literature identifies partner management as one of the main challenges in syphilis control, contributing to the persistence of transmission chains and recurrent maternal infections (32,33). Barriers such as lack of partner engagement with health services, stigma associated with STIs, weaknesses in communication between users and professionals, and organizational limitations within the care network significantly contribute to the persistence of this problem, especially in vulnerable urban contexts (33-35).

It is also noteworthy that the spatiotemporal analysis demonstrated that the distribution of GS is not random, presenting clusters that persist throughout the analyzed years, indicating that the disease does not spread uniformly across the region. This pattern largely reflects historical inequalities linked to socially vulnerable groups, such as women who self-identify as mixed-race, who tend to reside in peripheral urban areas and have less timely access to health services. Studies using similar methods, such as KDE and spatiotemporal models, have already shown that these clusters tend to persist over time, especially in the absence of targeted interventions (15,35,36). Therefore, these findings reinforce the importance of systematically incorporating spatial analysis into epidemiological surveillance as a tool to identify priority areas and better allocate available resources.

Moreover, the upward trend that persisted even after the most critical period of the SARS-CoV-2 pandemic suggests that the effects of disruptions in health care were long-lasting. Recent research indicates that the pandemic exacerbated pre-existing problems in prenatal care, such as reduced number of consultations, delayed diagnoses, and decreased active search for pregnant women, particularly in regions with greater socioeconomic vulnerability (37,38). Thus, the lack of proportional recovery in subsequent years highlights the need to develop specific strategies to reestablish adequate care, going beyond simply resuming previous practices (27,37).

Overall, the results indicated that GS is a condition strongly influenced by structural and organizational factors. Therefore, it is necessary to move beyond isolated interventions and implement integrated strategies. These actions should include more localized surveillance, improvements in prenatal care quality, expanded access to rapid and confirmatory testing, and innovative approaches to partner care. The combination of these measures is essential to ensure a consistent reduction in GS (27,39,40).

Finally, it is important to highlight that the regional focus adopted in this study helps fill important gaps in the literature on GS in the Brazilian Amazon. The spatiotemporal analysis, especially at the metropolitan level, allows for understanding local dynamics that are often not captured in broader analyses, providing valuable information for planning more equitable, fair, and effective policies in the region. GS presents a heterogeneous spatiotemporal distribution, influenced by structural, social, and organizational factors of health services. The persistence of case clusters and late diagnosis highlights weaknesses in prenatal care, timely diagnosis, and adequate management of pregnant women and their sexual partners.

## Data Availability

All data generated or analyzed during this study are included in this published article.

## Supplementary Materials

Supplementary Material Click here to view [pdf].

## References

[1] Brasil, Ministério da saúde (2025). Secretaria de Vigilancia em Saúde e Ambiente. Boletim epidemiológico - sífilis 2025.

[B02] Ramos AM, Ramos TJM, Costa ILOF, Reis APO, Lima SBA, Paiva DSBS (2022). Perfil epidemiológico da sífilis em gestantes no Brasil [in Portuguese]. Rev Eletr Acerv Saude.

[3] Melz M, de Souza AQ (2022). Assistência de enfermagem e a sífilis congênita: revisão integrativa [in Portuguese]. Rev Saude Dom Alb.

[4] Portela TJA, Silva MAM, Araújo DG, Freitas CASL, Mazza VA, Florêncio RS (2025). Vulnerabilidades em saúde para transmissão vertical da sífilis: situação programática dos serviços da atenção primária em uma região de saúde no Brasil [in Portuguese]. Rev Eletr Enferm.

[5] Leal MC, Esteves-Pereira AP, Viellas EF, Domingues RMSM, da Gama SGN da (2020). Prenatal care in the Brazilian public health services. Rev Saude Publica.

[6] Silva GIS (2025). Análise de séries temporais e distribuição espacial dos casos e indicadores epidemiológicos e operacionais da sífilis em gestantes no Maranhão, período 2009-2023 [in Portuguese]. Rev Saber Digital.

[7] Paschoalotto MAC, Passador JL, Passador CS, Endo GY (2022). Regionalização da saúde no Brasil: desigualdades socioeconômicas e na performance em saúde [in Portuguese]. Gest Regional.

[8] Fausto MCR, Giovanella L, Lima JG, Cabral LM da S, Seidl H (2022 Apr). Sustentabilidade da Atenção Primária è Saúde em territórios rurais remotos na Amazônia fluvial: organização, estratégias e desafios. Ciênc saúde coletiva.

[9] Barros ICA, Sousa CCM, da Silva NRF, Mascarenhas MDM (2024). Characterization of cases and epidemiological and operational indicators of leprosy: analysis of time series and spatial distribution, Piauí state, Brazil, 2007-2021. Epidemiol Serv Saude.

[10] BRASIL (2019). Regionalização da Saúde - posicionamento e orientações.

[11] Astolfo S, Andrade ACS, Kehrig RT (2024). Temporal analysis and spatial distribution of acquired syphilis in the state of Mato Grosso, Brazil, 2010-2021: an ecological study. Epidemiol Serv Saude.

[12] Ramos RSPS, Ramos VO (2021). Spatial analysis as a tool for identification of priority intervention areas for syphilis prevention [in Portuguese]. Cienc Saude Colet.

[13] Instituto Brasileiro de Geografia e Estatística (IBGE) População estimada 2025 [internet]. cidades@.

[14] Instituto Brasileiro de Geografia (IBGE) ĺndice do Desenvolvimento Humano Municipal (IDHM) 2010. cidades@.

[15] da Silva TPR, Schreck RSC, de Oliveira DCB, Mascarenhas LV, Luvisaro BMO, Camargo BTS (2024). Spatial and trend analysis of gestational syphilis cases in Brazil from 2011 to 2020: an ecological study. BMC Public Health.

[16] Medeiros JAR, Yamamura M, da Silva ZP, Domingues CSB, Waldman EA, Chiaravalloti-Neto F (2022). Spatiotemporal dynamics of syphilis in pregnant women and congenital syphilis in the state of São Paulo, Brazil. Sci Rep.

[17] França APM, de Sousa CM, de Lima MSA, Fonseca RRS, Laurentino RV, Monteiro JC (2024). High prevalence of syphilis among young pregnant women in the Brazilian Amazon: a cross-sectional study in Belém. Pathogens.

[18] Paixão ES, Carrol O, Rodrigues LC, de Oliveira ALS, Cardoso LSM, Ribeiro-Silva RC (2025). Syphilis exposure during pregnancy and childhood hospital admissions in Brazil. JAMA Network Open.

[B19] Barbosa PEB, Lima HC, Dornelas JPAP, Silva VDS, Cartaxo HB (2023). The relationship between prenatal care and adherence to treatment for Congenital Syphilis from 2018 to 2021. Res Soc Dev.

[B20] Santiago PBM, Meneses MES, Pereira LLL, Meneses MFS, Silva PA, Mariz FNC (2025). Maternal syphilis in the Federal District, Brazil: a five-year analysis of notified cases (2019-2023). Front Epidemiol.

[21] Aragón M, de Lannoy LH, Gaspar PC, da Silva APB, Coelho RA, Barreira D (2025). Syphilis in pregnant women living with HIV in Brazil: implications for maternal and child health. BMC Public Health.

[22] WHO (World Health Organization) (2021). Global progress report on HIV, viral hepatitis and sexually transmitted infections, 2021.

[B23] Pavinati G, de Lima LV, Stolarz MF, Gomes MF, Turquino SNS, Magnabosco GT (2025). Temporal analysis of gestational and congenital syphilis indicators in Brazil: toward the elimination of vertical transmission by 2030?. Rev Bras Epidemiol.

[B24] Salomà S, Cambriglia MD, Montesano G, Capasso L, Raimondi F (2024). Congenital syphilis: a re-emerging but preventable infection. Pathogens.

[25] Mareco TCS, Lima TGFMS, Ramos MNP, Dos Santos MMSL, da Silva JA, Ramos MNP (2023). Analyzing a national health surveillance strategy to reduce mother-to-child transmission of syphilis: the case of Brazilian investigation committees. IJID Reg.

[26] Organização Pan Americana da Saúde (2025). Reunião regional convoca países das Américas a priorizar ações de testagem e tratamento da sífilis.

[27] Brasil (2022). Protocolo clínico e diretrizes terapêuticas para atenção integral ès pessoas com infecções sexualmente transmissíveis.

[28] Echegaray F, Hernandez CJ, Sundar KG, Yang LZ, Cambou MC, Segura ER (2025). Repercussions of the COVID- 19 pandemic on maternal and congenital syphilis in South Brazil: a time series analysis 2010-2022. BMC Infect Dis.

[29] Miranda AE, Santos PC, Coelho RA, Pascom ARP, Lannoy LH, Ferreira ACG (2023). Perspectives and challenges for mother-to-child transmission of HIV, hepatitis B, and syphilis in Brazil. Front Public Health.

[30] Fuertes-Bucheli JF, Buenaventura-Alegría DP, Rivas-Mina AM, Pacheco-López R (2024). Congenital syphilis prevention challenges, Pacific Coast of Colombia, 2018-2022. Emerg Infect Dis.

[31] Costa IB, Pimenta IDSF, Aiquoc KM, Oliveira ÂGRC (2024). Congenital syphilis, syphilis in pregnancy and prenatal care in Brazil: an ecological study. PLoS One.

[32] Fernandes LPMR, Oliveira CNT, Brito BB, de Melo FF, Souza CL, Oliveira MV (2022). Prevalence and factors associated with non-adherence to therapy among partners of pregnant women with syphilis in a city of northeastern Brazil. World J Obstet Gynecol.

[33] Lopes IMD, Alves GL, Pimentel JVA, Silva JRS, Reis FP, Lima SO (2025). Sífilis gestacional e congênita: adesão ao tratamento e seguimento em uma maternidade do Nordeste brasileiro [in Portuguese]. Temas Saúde.

[B34] Rocha CAG, Ribeiro BL, da Silva MEA, Pimentel MS, dos Santos TS, Almeida MS (2025). Atenção primária è saúde no enfrentamento de sífilis gestacional e congênita: uma revisão integrativa [in Portuguese]. Arq Cienc Saude Unipar.

[35] Dantas JC, Marinho CSR, Pinheiro YT, Ferreira MÂF, da Silva RAR (2023). Spatial distribution of gestational syphilis in Brazil: socioeconomic and health services inequalities. Am J Trop Med Hyg.

[36] Kulldorff M (2021). SaTScan™ user guide for spatial analysis. Boston (MA): Harvard Medical School.

[37] Pinheiro YT, da Silva RAR (2022). Has the COVID-19 Pandemic Affected the epidemiology of syphilis in Brazil?. Rev Bras Ginecol Obstet.

[38] Chmielewska B, Barratt I, Townsend R, Kalafat E, van der Meulen J, Gurol-Urganci I (2021). Effects of the COVID-19 pandemic on maternal and perinatal outcomes: a systematic review and meta-analysis. Lancet Glob Health.

[39] WHO (World Health Organization) (2022). Global health sector strategies on HIV, viral hepatitis and sexually transmitted infections 2022-2030.

[40] Cope AB, Bernstein KT, Matthias J, Rahman M, Diesel JC, Pugsley RA (2022). Effectiveness of syphilis partner notification after adjusting for treatment dates, 7 jurisdictions. Sex Transm Dis.

