# Supplementary material for: Spatiotemporal heterogeneity of gestational syphilis in the 1st Health Region of Pará in the Brazilian Amazon
Source: Braz J Med Biol Res. 2026 Jul 3;59:e15574. doi: 10.1590/1414-431X2026e15574 (PMC13331249; doi:10.1590/1414-431X2026e15574)
Supplement: Supplementary Material [file 1414-431X-bjmbr-59-e15574-suppl.pdf]

Table S1. Sociodemographic description of gestational syphilis cases according to municipality. Belém, PA, Brazil, 2019–2024 (n=5,607).

	Ananindeua		Belém		Benevides		Marituba		Santa Bárbara		P*	Total	
	N	%	N	%	N	%	N	%	N	%		N	%
Year of notification													
2019	123	14.2	336	8.3	15	12.2	91	17.4	5	12.2	<0.001	570	10.2
2020	122	14.1	324	8.0	9	7.3	63	12.0	6	14.6		524	9.3
2021	173	20.0	489	12.1	17	13.8	108	20.7	5	12.2		792	14.1
2022	149	17.2	815	20.1	25	20.3	92	17.6	12	29.3		1093	19.5
2023	156	18.1	955	23.5	34	27.6	92	17.6	5	12.2		1242	22.2
2024	141	16.3	1137	28.0	23	18.7	77	14.7	8	19.5		1386	24.7
Pregnant woman													
1st trimester	128	14.8	758	18.7	42	34.1	120	22.9	21	51.2	<0.001	1069	19.1
2nd trimester	149	17.2	700	17.3	53	43.1	113	21.6	14	34.1		1029	18.4
3rd trimester	496	57.4	2430	59.9	25	20.3	282	53.9	6	14.6		3239	57.8
Gestational age unknown	91	10.5	168	4.1	3	2.4	8	1.5	0	0.0		270	4.8
Race/Color													
White	47	5.9	340	8.9	14	11.8	46	10.6	10	25.0	<0.001	457	8.8
Black	57	7.2	235	6.2	15	12.6	41	9.5	5	12.5		353	6.8
Yellow	5	0.6	20	0.5	0	0.0	5	1.2	1	2.5		31	0.6
Mixed-race	682	86.0	3216	84.2	89	74.8	341	78.8	24	60.0		4352	83.6
Indigenous	2	0.3	7	0.2	1	0.8	0	0.0	0	0.0		10	0.2
Education													
Illiterate/No schooling	15	1.9	127	3.2	2	1.6	10	1.9	2	5.9	<0.001	156	2.8
Complete 4th grade of Elementary School	115	14.6	860	21.4	16	13.0	87	167	9	26.5		1087	19.8
Complete Elementary School	193	24.5	890	22.2	29	23.6	104	20.0	8	23.5		1224	22.3
Complete High School	277	35.1	1241	30.9	33	26.8	129	24.8	13	38.2		1693	30.9
Higher Education	13	1.6	74	1.8	1	0.8	6	1.2	0	0.0		94	1.7
Unknown	176	22.3	818	20.4	42	34.1	185	35.5	2	5.9		1223	22.3
Not applicable	0	0.0	1	0.0	0	0.0	0	0.0	0	0.0		1	0.0
Municipality of residence													
Ananindeua	700	81.0	452	11.1	0	0.0	35	6.7	0	0.0	<0.001	1187	21.2
Belém	80	9.3	3468	85.5	0	0.0	14	2.7	0	0.0		3562	63.5
Benevides	23	2.7	40	1.0	123	100.0	46	8.8	0	0.0		232	4.1
Marituba	55	.	84	2.1	0	0.0	415	79.3	0	0.0		554	9.9
Santa Bárbara do Pará	6	0.7	12	0.3	0	0.0	13	2.5	41	100.0		72	1.3
Zone													
Urban	788	99.0	3900	98.6	101	83.5	516	98.7	22	57.9	–	5327	98.0
Rural	7	0.9	42	1.1	19	15.7	5	1.0	16	42.1		89	1.6
Peri-urban	1	0.1	14	0.4	1	0.8	2	0.4	0	0.0		18	0.3
Municipality where prenatal care is provided													
Unknown/Other municipalities	122	14.1	1113	27.4	1	0.8	27	5.2	4	9.8	<0.001	1267	22.6
Ananindeua	593	68.6	262	6.5	0	0.0	35	6.7	0	0.0		890	15.9
Belém	85	9.8	2588	63.8	0	0.0	18	3.4	0	0.0		2691	48.0
Benevides	17	2.0	25	0.6	122	99.2	44	8.4	0	0.0		208	3.7
Marituba	44	5.1	58	1.4	0	0.0	387	74.0	0	0.0		489	8.7
Santa Bárbara do Pará	3	0.3	10	0.2	0	0.0	12	2.3	37	90.2		62	1.1
	M	SD	M	SD	M	SD	M	SD	M	SD		M	SD
Age	23.85	5.57	23.94	5.82	24.43	5.71	23.11	5.31	21.22	4.80	0.001	23.84	5.74

N: absolute frequency; %: relative frequency; M: mean; SD: standard deviation. *P-value for the Fisher's exact test for categorical variables and the Kruskal-Wallis test for numerical variables.

Table S2. Clinical characteristics and diagnoses of gestational syphilis cases according to municipality. Belém, PA, Brazil, 2019-2024 (n=5,607).

	Ananindeua		Belém		Benevides		Marituba		Santa Bárbara		P*	Total	
	N	%	N	%	N	%	N	%	N	%		N	%
Clinical classification													
Primary	299	44.6	1128	41.7	25	28.7	113	22.2	14	38.9	<0.001	1579	39.4
Secondary	23	3.4	207	7.6	9	10.3	24	4.7	2	5.6		265	6.6
Tertiary	206	30.7	686	25.3	36	41.4	284	55.7	11	30.6		1223	30.5
Latent	142	21.2	686	25.3	17	19.5	89	17.5	9	25.0		943	23.5
Prenatal non-treponemal test													
Reactive	596	76.3	3723	93.0	75	66.4	342	65.6	26	66.7	–	4762	87.3
Non-reactive	47	6.0	132	3.3	8	7.1	4	0.8	2	5.1		193	3.5
Not performed	138	17.7	148	3.7	30	26.5	175	33.6	11	28.2		502	9.2
Prenatal treponemal test													
Reactive	620	77.9	2516	70.2	93	77.5	348	67.4	31	75.6	–	3608	71.4
Non-reactive	57	7.2	144	4.0	16	13.3	35	6.8	7	17.1		259	5.1
Not performed	119	14.9	923	25.8	11	9.2	133	25.8	3	7.3		1189	23.5
	M	SD	M	SD	M	SD	M	SD	M	SD		M	SD
VDRL titer result	17	36	36	104	347947	2783031	35	182	14	17	<0.001	5513	349658

N: absolute frequency; %: relative frequency; M: mean; SD: standard deviation; VDRL: Venereal Disease Research Laboratory. *P-value for the Fisher's exact test for categorical variables and the Kruskal-Wallis test for numerical variables.

Table S3. Description of maternal and partner treatment in gestational syphilis cases according to municipality. Belém, PA, Brazil, 2019–2024 (n=5,607).

	Ananindeua		Belém		Benevides		Marituba		Santa Bárbara		P*	Total	
	N	%	N	%	N	%	N	%	N	%		N	%
Treatment regimen for the pregnant woman													
Benzathine penicillin G 2.4 million IU	219	30.2	754	18.9	20	16.5	113	21.7	8	19.5	<0.001	1114	20.6
Benzathine penicillin G 4.8 million IU	13	1.8	100	2.5	4	3.3	19	3.7	2	4.9		138	2.6
Benzathine penicillin G 7.2 million IU	388	53.5	2751	68.9	92	76.0	375	72.1	31	75.6		3637	67.4
Other regimen	26	3.6	59	1.5	3	2.5	2	0.4	0	0.0		90	1.7
Not performed	79	10.9	326	8.2	2	1.7	11	2.1	0	0.0		418	7.7
Partner treated concomitantly with the pregnant woman													
Yes	274	44.1	1280	40.4	50	51.0	150	45.2	20	55.6	0.021	1774	41.7
No	347	55.9	1885	59.6	48	49.0	182	54.8	16	44.4		2478	58.3
Treatment regimen prescribed to the partner													
Benzathine penicillin G 2.4 million IU	106	19.5	272	9.1	11	12.0	67	21.8	9	25.0	<0.001	465	11.7
Benzathine penicillin G 4.8 million IU	8	1.5	61	2.0	2	2.2	11	3.6	1	2.8		83	2.1
Benzathine penicillin G 7.2 million IU	147	27.1	1249	41.6	42	45.7	77	25.1	11	30.6		1526	38.4
Other regimen	20	3.7	23	0.8	1	1.1	0	0.0	0	0.0		44	1.1
Not performed	262	48.3	1394	46.5	36	39.1	152	49.5	15	41.7		1859	46.7
Reason for non-treatment of the partner													
Partner no longer had contact with the pregnant woman	114	23.6	431	24.3	72	66.1	25	44.6	5	25.0	<0.001	647	26.5
Partner was not notified/called to the HU for treatment	51	10.5	252	14.2	4	3.7	5	8.9	0	0.0		312	12.8
Partner was notified/called to the HU for treatment, but did not attend	24	5.0	141	7.9	17	15.6	7	12.5	5	25.0		194	7.9
Partner was notified/called to the HU but refused treatment	0	0.0	29	1.6	0	0.0	0	0.0	1	5.0		30	1.2
Partner with non-reactive serology	46	9.5	88	5.0	3	2.8	7	12.5	3	15.0		147	6.0
Other reason	249	51.4	836	47.0	13	11.9	12	21.4	6	30.0		1116	45.6

N: absolute frequency; %: relative frequency; IU: international unit; HU: health unit; M: mean; SD: standard deviation. *P-value for the Fisher's exact test for categorical variables and the Kruskal-Wallis test for numerical variables.
